# Uridine Alleviates Sepsis-Induced Acute Lung Injury by Inhibiting Ferroptosis of Macrophage

**DOI:** 10.3390/ijms24065093

**Published:** 2023-03-07

**Authors:** Kai Lai, Congkuan Song, Minglang Gao, Yu Deng, Zilong Lu, Ning Li, Qing Geng

**Affiliations:** Department of Thoracic Surgery, Renmin Hospital of Wuhan University, Wuhan 430060, China

**Keywords:** uridine, acute lung injury, ferroptosis, macrophage, oxidative stress

## Abstract

Uridine metabolism is extensively reported to be involved in combating oxidative stress. Redox-imbalance-mediated ferroptosis plays a pivotal role in sepsis-induced acute lung injury (ALI). This study aims to explore the role of uridine metabolism in sepsis-induced ALI and the regulatory mechanism of uridine in ferroptosis. The Gene Expression Omnibus (GEO) datasets including lung tissues in lipopolysaccharides (LPS) -induced ALI model or human blood sample of sepsis were collected. In vivo and vitro, LPS was injected into mice or administered to THP-1 cells to generate sepsis or inflammatory models. We identified that uridine phosphorylase 1 (UPP1) was upregulated in lung tissues and septic blood samples and uridine significantly alleviated lung injury, inflammation, tissue iron level and lipid peroxidation. Nonetheless, the expression of ferroptosis biomarkers, including SLC7A11, GPX4 and HO-1, were upregulated, while lipid synthesis gene (ACSL4) expression was greatly restricted by uridine supplementation. Moreover, pretreatment of ferroptosis inducer (Erastin or Era) weakened while inhibitor (Ferrostatin-1 or Fer-1) strengthened the protective effects of uridine. Mechanistically, uridine inhibited macrophage ferroptosis by activating Nrf2 signaling pathway. In conclusion, uridine metabolism dysregulation is a novel accelerator for sepsis-induced ALI and uridine supplementation may offer a potential avenue for ameliorating sepsis-induced ALI by suppressing ferroptosis.

## 1. Introduction

Acute lung injury (ALI) is a severe respiratory disease characterized by diffused non-cardiogenic pulmonary edema due to alveolar damage in pathological manifestation and featured irreformable hypoxemia and respiratory distress in clinical performance [1]. For lack of effective treatment options currently, ALI often develops into its extreme form acute respiratory distress (ARDS), a clinical life-threatening syndrome with high mortality globally [2]. In pathogenesis, ALI can be caused by a variety of pathogenic factors through direct damage or indirect injury imposed by hyper inflammation [3]. Among these, sepsis is the primary risk factor for leading ALI, as the lung is extremely susceptible to the grave immune response stimulated by sepsis during the multiorgan dysfunction stage [4,5]. However, to date, the exact mechanism underpinning sepsis-induced ALI remains obscure and effective agents are exceedingly limited [6,7]. Therefore, it is imperative to dig out the pathogenesis of sepsis-induced ALI and try to discover effective interventions to improve outcomes for patients who are struggling with sepsis.

Uridine, a pyrimidine nucleoside, is composed of uracil and ribose and employed to synthesize pyrimidine nucleotide [8]. Uridine metabolism, including anabolism and catabolism, are a series of fine controlled processes and intimately regulated by a spectrum of enzymes [9]. Increasing studies have shown that uridine, the most abundant nucleoside in human blood, affects multiple physiological processes, genetic material and glycogen synthesis [10]. Additionally, the disturbance of uridine metabolism has been implicated in the progression of a set of disease courses [11,12,13]. It is conceivable theoretically that the elaborately regulated processes of uridine metabolism are inseparable from its essential role in modulating pathophysiological processes. As a matter of fact, emerging researches have reported that uridine exerts anti-inflammation [14,15], anti-fibrosis [16], antioxidation [17,18,19] and anti-aging functions [20]. For example, Irina B. Krylova1 discovered that uridine treatment attenuated myocardial ischemic-reperfusion injury via antioxidation by activating ATP-dependent potassium (mitoKATP) channel [21]. Nevertheless, in a cross-species metabolomic study, higher concentration of uridine was identified to exist in blood of younger counterparts and supplementation of uridine was validated to recover regenerative capability of older tissues and promoted the proliferation of stem cells [22]. Moreover, uridine exhibited potent anti-inflammatory actions during endotoxemia in murine model [23]. However, the landscape of uridine metabolism in sepsis-induced ALI is largely unknown and the other mechanisms underpinning the protective effects of uridine deserve to be explored.

Ferroptosis is a newly discovered cell death form, defined by iron-dependent lipid peroxidation leading to membrane destruction and cellular contents outflowing, eventually causing cell death [24]. Mounting evidence has demonstrated that ferroptosis is implicated in various pathophysiological processes and involved in various of human diseases, including ALI [25], acute myocardial infarction [26], tumorigenesis [27], acute kidney injury [28], etc. Nuclear factor erythroid 2-related factor 2 (NFE2L2, or Nrf2), a key modulator for combating cellular oxidative stress, is well reported to play a critical role in regulating ferroptosis [29]. Given the antioxidant property of uridine, it is conceivable and desirable to probe into the relationship between uridine metabolism and ferroptosis in sepsis-induced ALI.

Herein, we discovered that uridine metabolism was disturbed in sepsis-induced ALI. In addition, uridine supplementation mitigated sepsis-induced ALI by inhibiting macrophage ferroptosis via activating Nrf2 pathway and inhibiting Acyl-CoA synthetase long-chain family member 4 (ACSL4) expression. Overall, uridine is identified to be an endogenous anti-inflammatory metabolite, and exogenous supplementation of uridine could be a promising therapeutic intervention for sepsis-induced ALI.

## 2. Results

### 2.1. Uridine Metabolism Homeostasis Was Disturbed in Sepsis-Induced ALI

**Figure 1:**
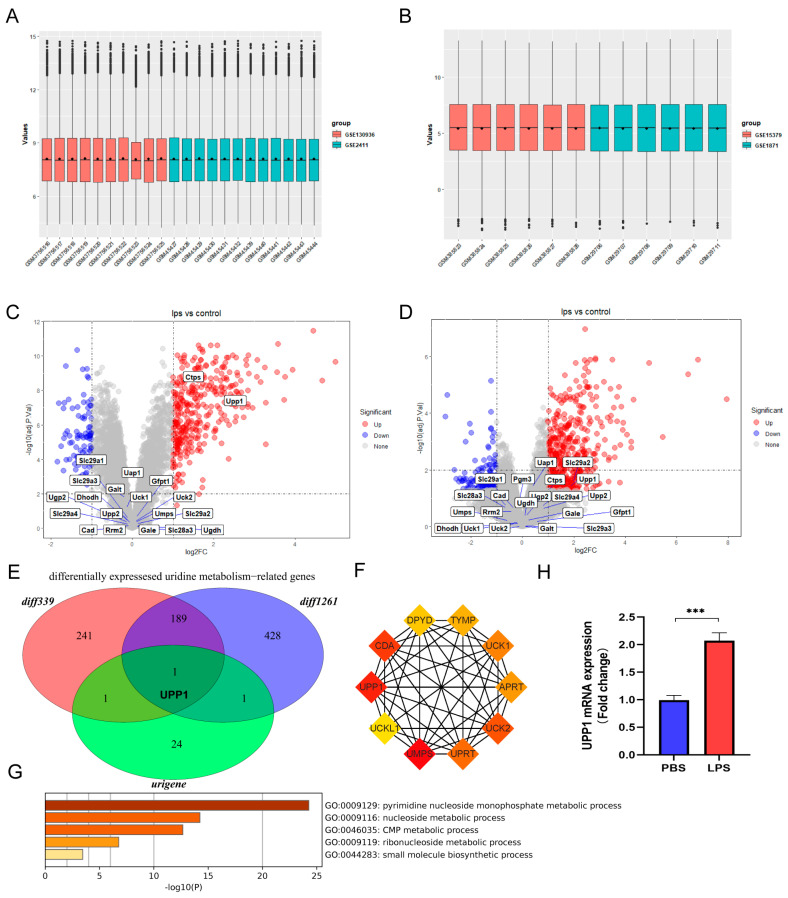
Identification and validation of dysregulated uridine metabolism in lung tissues of sepsis-induced ALI mice. (**A**) Gene expression level of the integrated datasets with same platform GPL339 after removing batch effects. (**B**) Gene expression level of the integrated datasets with same platform GPL1261 after removing batch effects. (**C**) The volcano plot of the differentially expressed genes between sepsis and control group in the integrated dataset of the same platform GPL339 and annotated with uridine metabolism-related genes. (**D**) The volcano plot of the differentially expressed genes between sepsis and control group in the integrated dataset of the same platform GPL1261 annotated with uridine metabolism-related genes. (**E**) Venn diagram of the differentially expressed genes in the integrated datasets in same GPL339 and same GPL1261 and uridine metabolism-related genes. The red circle and blue circle represent the differentially expressed genes in GPL339 and GPL1261, annotated diff339 and diff1261, respectively. The green circle means uridine metabolism-related genes, annotated urigene. (**F**) The PPI network shows the top 10 interacted protein of uridine phosphorylase 1 (UPP1). Each box represents one protein and the darker the color is, the more inseparable the interaction with other proteins are. (**G**) The UPP1 mRNA level of lung tissues between sepsis-induced ALI and control group. (**H**) Relative mRNA levels of IL6, TNFα and IL1β in lung tissue in the indicated group. n = 6 mice per group. (Data are presented as Mean ± SD, *** *p* < 0.001 compared with indicated group).

To explore the role of uridine metabolism in sepsis-induced ALI, we collected uridine metabolism-related genes and downloaded microarray datasets of sepsis-induced ALI. The cohorts with same platform were integrated into a larger cohort by removing batch effects. As shown in Figure 1A,B, the general expression value in each integrated GEO cohort demonstrated that batch effects were removed. Then, differentially expressed analysis was performed for each integrated GEO cohort and the differentially expressed genes were intersected with uridine metabolism-related genes. Interestingly, there were both two differentially expressed uridine metabolism-related genes in GPL339 and GPL1261, and we found that uridine phosphorylase 1 (UPP1) was the only differentially expressed uridine metabolism-related gene in sepsis-induced lung tissue compared to control (Figure 1C–E). UPP1 is well known for functioning as key enzyme in catalyzing uridine to uracil and ribose-1-phosphate. To confirm the function of UPP1 further, we constructed the PPI network for UPP1 and discovered these genes were closely related to uridine metabolism (Figure 1F,G). Moreover, we detected the mRNA level of UPP1 in lung tissues of sepsis-induced ALI. Consistently, UPP1 was validated to be significantly upregulated in sepsis-induced ALI (Figure 1H). Taken together, these data suggested that uridine metabolism in lung tissues was interfered in sepsis-induced ALI.

### 2.2. Uridine Supplementation Alleviated Sepsis-Induced ALI and Inflammation of Lung Tissue

**Figure 2:**
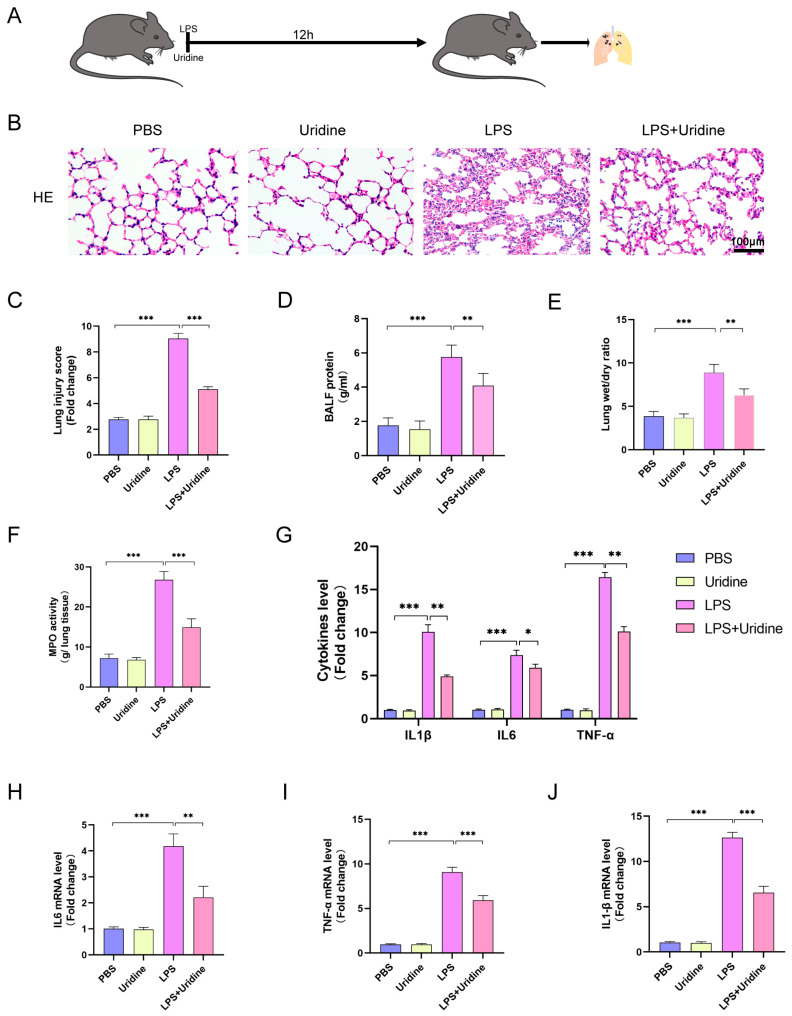
Uridine supplementation significantly attenuated sepsis-induced ALI in vivo. (**A**) Schematic diagram of animal experiment. (**B**,**C**) Representative images of H&E staining of mouse lung tissue at 12 h after LPS injection and semiquantitative lung injury scores in groups were analyzed based on the pathological section results. (**D**) BALF protein level in the indicated group. (**E**) Lung wet-to-dry weight ratio was determined in the indicated group. (**F**) The myeloperoxidase (MPO) activity of lung tissue in the indicated group. (**G**) ELISA for IL1β, IL6 and TNFα in lung tissue in the indicated group. (**H**–**J**). Relative mRNA levels of IL6, TNFα and IL1β in lung tissue in the indicated group. n = 6 mice per group (Data are presented as Mean ± SD, * *p* < 0.05, ** *p* < 0.01, *** *p* < 0.001 compared with indicated group).

In the first part, we discovered UPP1 was upregulated after LPS stimulation. Considering the function of UPP1 proposed to decrease uridine level, we hypothesized that maintaining uridine metabolism homeostasis through uridine supplementation could benefit sepsis-induced ALI. To address the assumption and investigate the function of uridine, sepsis-induced ALI model was established by LPS injection intraperitoneally and uridine treatment at the same time (Figure 2A). As expected, compared to the LPS group, uridine treatment attenuated sepsis-induced ALI pathological changes, as manifested by pulmonary hemorrhage, interstitial edema and thickening of the alveolar wall in HE staining (Figure 2B). Furthermore, LPS stimulation markedly increased the lung injury score, while uridine significantly reversed the damage (Figure 2C). In addition, the protein level in BALF and lung edema extent were also lightened in uridine treatment group compared with LPS stimulation alone group (Figure 2D,E). For inflammatory cell infiltration level, we detected the activity of MPO, a biomarker for neutrophils, and found that MPO activity was significantly higher in the LPS group than in the control group, however, uridine treatment greatly reversed this situation (Figure 2F). Similarly, uridine supplementation markedly prevented the inflammatory response in lung tissues, as reflected by the reduced protein and mRNA level of IL-6, TNF-α and IL-1β (Figure 2G–J). Altogether, these data unveiled that uridine supplementation could inhibit the extent of lung damage as well as inflammation response in sepsis-induced ALI in vivo.

### 2.3. Uridine Inhibited Ferroptosis in Lung Tissue Dependent on Nrf2 Activation and ACSL4 Inhibition

**Figure 3:**
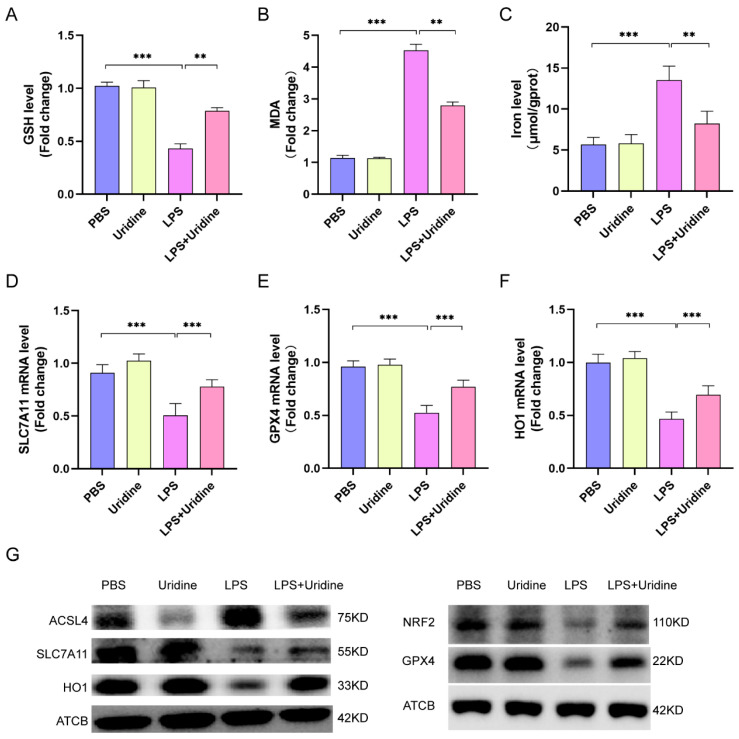
Uridine supplementation inhibited ferroptosis in murine lung via upregulating Nrf2-dependent antioxidant pathways and downregulating ACSL4 expression. (**A**–**C**) Relative GSH, MDA and iron levels in murine lung tissue. (**D**–**F**) Relative mRNA expression of SLC7A11, GPX4 and HO1 in murine lung tissue. (**G**) Western blots for NRF2, SLC7A11, HO1, ACSL4, GPX4 and ATCB (β-actin) in murine lung tissues. n = 6 per group. (Data are presented as Mean ± SD, ** *p* < 0.01, *** *p* < 0.001 compared with indicated group).

Previous study has reported that uridine could mitigate lipid peroxidation in myocardial ischemic/reperfusion model [21]. Additionally, the connection between ferroptosis and uridine is enlightened by the discovery that the pyrimidine biosynthesis enzyme, dihydroorotate dehydrogenase (DHODH), is a ferroptosis defender [30]. However, the relationship between ferroptosis (iron-dependent lipid peroxidation) and uridine metabolism in sepsis-induced ALI is still largely unknown. To address the role of uridine in ferroptosis, the level of glutathione (GSH, a reductive agent for antioxidation), lipid peroxidation products MDA and tissue iron level were assessed. It was found that LPS stimulation significantly reduced the GSH level, while uridine treatment reversed this situation and increased GSH extent in lung tissue (Figure 3A). In contrast, the MDA level and tissue iron were decreased after uridine supplementation (LPS+uridine) compared with LPS group (Figure 3B,C). Moreover, SLC7A11, functioning to exchange intracellular glutamate for extracellular cystine, and glutathione-dependent antioxidant enzyme GPX4 are two well accepted markers of ferroptosis [31]. Our results demonstrated that the protein and mRNA of SLC7A11 and GPX4 were both downregulated after LPS stimulation, however, uridine treatment greatly upregulated their expression (Figure 3D,E,G). It is well known that Nrf2 is an antioxidant response element, which could promote HO1, GPX4 and SLC7A11 expression to inhibit ferroptosis. Thus, we detected the expression of Nrf2/HO1 axis. As expected, uridine augmented HO1 expression via activating Nrf2 (Figure 3F,G). Additionally, ACSL4 is a key enzyme that regulates lipid synthesis, thus, to promote ferroptosis. Our result unveiled that uridine obviously weakened the expression of ACSL4, which was markedly raised in LPS group (Figure 3G). Conclusively, our results revealed that uridine inhibited the ferroptosis via activating Nrf2 pathway and suppressing ACSL4 expression in sepsis-induced ALI in vivo.

### 2.4. Uridine Supplementation Restrained Inflammation and Oxidative Stress in THP-1 Cells

**Figure 4:**
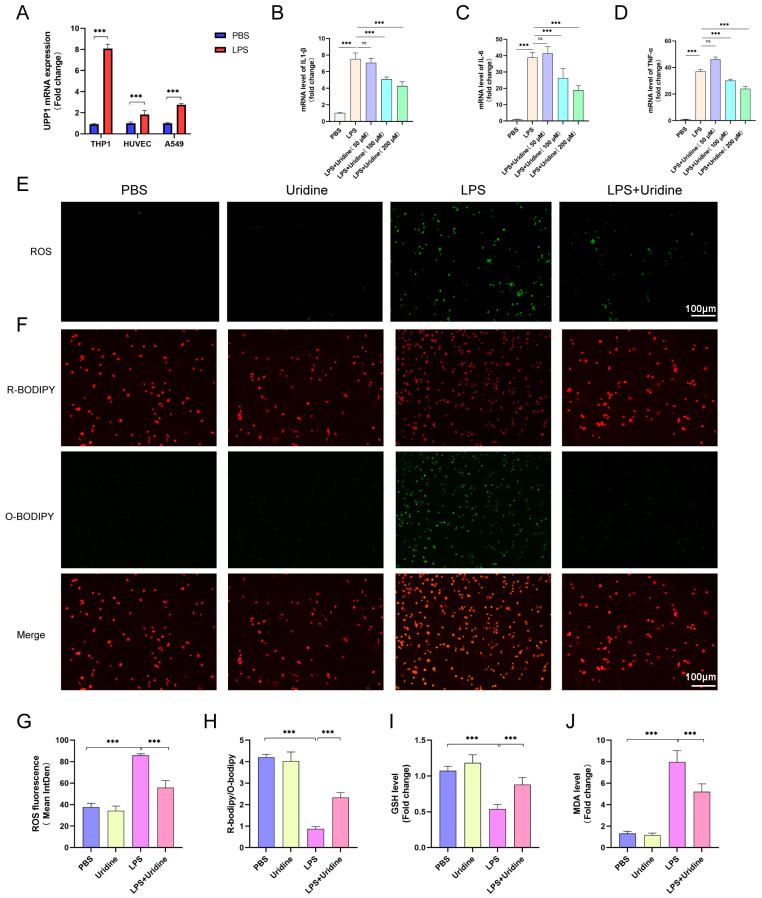
Uridine supplementation alleviated oxidative stress and lipid peroxidation in THP-1 cells. (**A**) Relative UPP1 mRNA expression levels after LPS (10 μg/mL) stimulation 8 h in THP-1, HUVEC and A549 cells. (**B**–**D**) Relative mRNA expression levels of IL1β, IL6 and TNFα in THP-1 cells after LPS stimulation with uridine supplementation in different concentration. LPS and uridine were almost simultaneously administrated. (**E**) Representative images of fluorescence probe for ROS in the indicated group in THP-1 cells after LPS stimulation with or without uridine (200 μM) supplementation. (**F**) Representative images of fluorescence probe for lipid peroxidation in the indicated group in THP-1 cells. (**G**) The statistical results for ROS. (**H**) The statistical results for lipid peroxidation. Green and red fluorescence represents oxidative and reduced lipid respectively. (**I**,**J**) Relative GSH and MDA level in THP-1 cells lysates. n = 6 per group, (Data are presented as Mean ± SD, *** *p* < 0.001 compared with indicated group, ns, no significance).

To determine which cell may benefit uridine supplementation most, we evaluated the UPP1 expression after LPS stimulation on three cell types, which are representatives for playing a vital role in the pathogenesis of sepsis-induced ALI. As shown in Figure 4A, UPP1 mRNA expression was dramatically enhanced in THP-1 cells (on behalf of alveolar macrophage) than other two cells. Therefore, THP-1 cells were selected for further experiments. Next, to ascertain the optimal concentration for uridine treatment and anti-inflammatory properties of uridine in vitro, we measured the mRNA level of IL-1β, IL-6 and TNF-α after LPS stimulation supplemented with different concentration of uridine. The results showed that the anti-inflammatory effects of uridine were fortified with the increase of concentration (Figure 4B–D). Eventually, we selected 200 μM uridine to treat THP-1 cells. In addition, we validated the antioxidative capacity of uridine in vitro. The ROS fluorescence intensity was observably promoted by LPS stimulation, which was significantly bated in LPS+uridine group (Figure 4E,G). Likewise, the C11-BODIPY fluorescence probe was used to determine cellular lipids and the probe will show green fluorescence when bound to oxidized lipids. It is quite clear from the results that oxidized lipids were dramatically increased after LPS stimulation, while uridine supplementation prevented that situation (Figure 4F,H). As a further verification, we examined the GSH and MDA level in THP-1 cells. Consistent with the results in lung tissue, uridine treatment also increased GSH level and lowered the MDA content in vitro (Figure 4I,J). These data suggested that uridine treatment stabilized cellular oxidation and reduction balance in vitro, thus, to exhibit anti-inflammatory effects.

### 2.5. Uridine Suppressed Ferroptosis in THP-1 Cells via Activating Nrf2 and Inhibiting ACSL4 Expression

**Figure 5:**
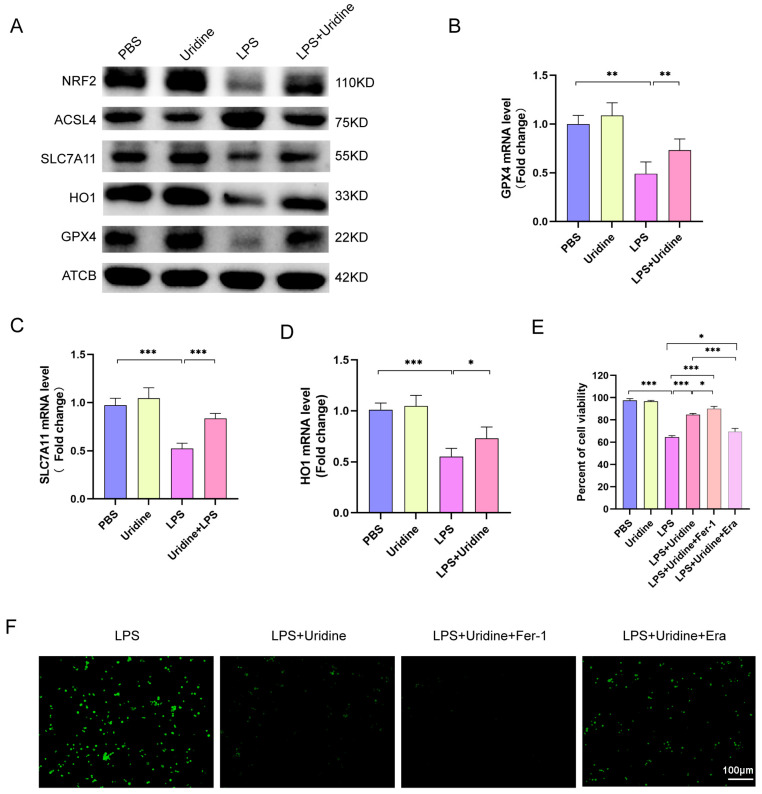
Uridine supplementation markedly suppressed ferroptosis in THP-1 cells after LPS stimulation. (**A**) Immunoblots for NRF2, ASCL4, SLC7A11, HO1, GPX4 and ATCB (β-actin) in the indicated group either treated with LPS (10 μg/mL) or uridine (200 μM) or both. (**B**–**D**) Relative mRNA expression of GPX4, SLC7A11 and HO1 in the indicated group. (**E**) Cell viability determined by CCK8 assay in the indicated group. Fer-1 (Ferrostatin-1, 5μM) and Era (Erastin, 5 μM) were treated 30 min after uridine supplementation. (**F**) Representative images of fluorescence probe for ROS. n = 6 per group (Data are presented as Mean ± SD, * *p* < 0.05, ** *p* < 0.01, *** *p* < 0.001 compared with indicated group).

To assess the relationship between uridine metabolism and ferroptosis in vitro, we treated uridine in THP-1 cells and examined the protein and mRNA expression of markers of ferroptosis. Similar results were observed in vitro just the same as in vivo. LPS administration significantly downregulated Nrf2, HO1, GPX4 and SLC7A11 expression in protein level, which were all greatly upregulated after uridine addition (Figure 5A). Furthermore, ACSL4 was decreased after uridine supplementation (Figure 5A). Meanwhile, we also discovered that the downregulated mRNA level of GPX4, SLC7A11 and HO1 by LPS were all restored after uridine addition (Figure 5B–D). Subsequently, the cell viability was examined by CCK8 for ferroptosis could lower the cell viability. As shown in Figure 5E, LPS stimulation markedly attenuated cell viability, while uridine supplementation abated the death effect of LPS. In addition, it was visualized that ferroptosis inhibitor ferrostatin-1 (Fer-1) application strengthened while ferroptosis inducer Erastin (Era) application weakened the protective effects of uridine (Figure 5E). Apart from cell viability, the inhibitory effect of uridine for regulating cellular ROS level was also adjusted by Fer-1 and Era (Figure 5F). Collectively, these data uncovered that uridine could inhibit ferroptosis in THP-1 cells.

### 2.6. Nrf2 Knockdown Abrogated the Ferroptosis Resistance Effect of Uridine

**Figure 6:**
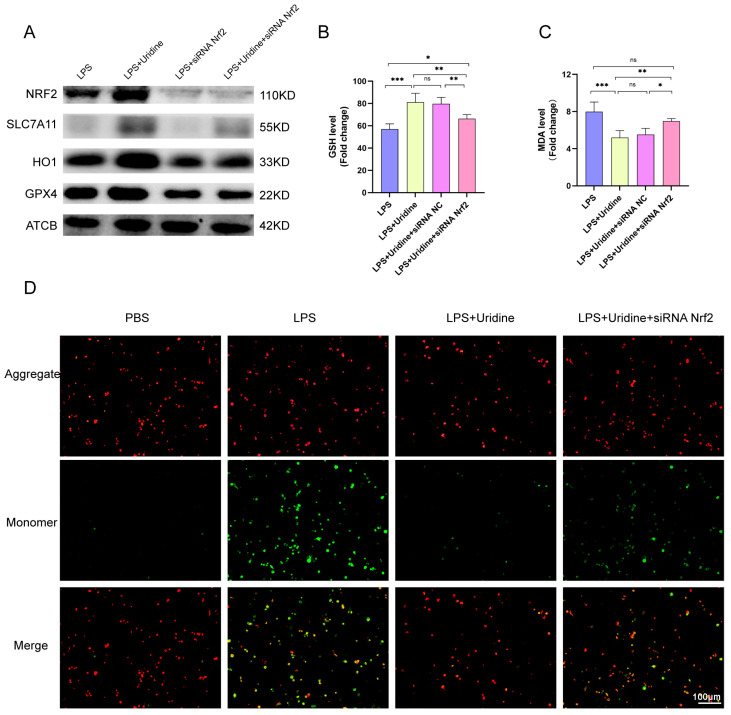
Nrf2 knockdown via siRNA abrogated the antioxidative effect of uridine. (**A**) Western blots for Nrf2, HO1, SLC7A11 and GPX4 protein of THP-1 cells in the indicated group. (**B**,**C**) Relative GSH and MDA levels in the indicated group. NC means negative control. (**D**) Representative images of fluorescence probe for mitochondrial membrane potential determined by JC-1. n = 6 per group. (Data are presented as Mean ± SD, * *p* < 0.05, ** *p* < 0.01, *** *p* < 0.001 compared with indicated group, ns, no significance).

To further validate the role of Nrf2 in the process of uridine combating ferroptosis, the Nrf2 was knockdown by siRNA and it successfully silenced Nrf2 expression in THP-1 cells (Figure 6A). At the same time, GPX4, SLC7A11 and HO1 expression were significantly decreased after Nrf2 silence, and the protective effect of uridine was subdued (Figure 6A). Otherwise, compared with LPS+uridine group, Nrf2 silence group had a lower cellular GSH level and higher MDA level (Figure 6B,C). Mitochondrial membrane potential decrease often indicates mitochondrial damage and mitochondrial oxidative stress occurrence, which is associated with ferroptosis. Thus, we measured the mitochondrial membrane potential by applying the JC-1 probe. When mitochondrial membrane is damaged, green fluorescence (monomer) accumulates. Consistently, uridine supplementation mitigated the mitochondrial damage, however, Nrf2 silence abrogated the protective effect (Figure 6D). These data further consolidated the role of Nrf2 in the ferroptosis resistant effect of uridine.

### 2.7. Dysregulated Uridine Metabolism Was Confirmed in Patients with Sepsis

**Figure 7:**
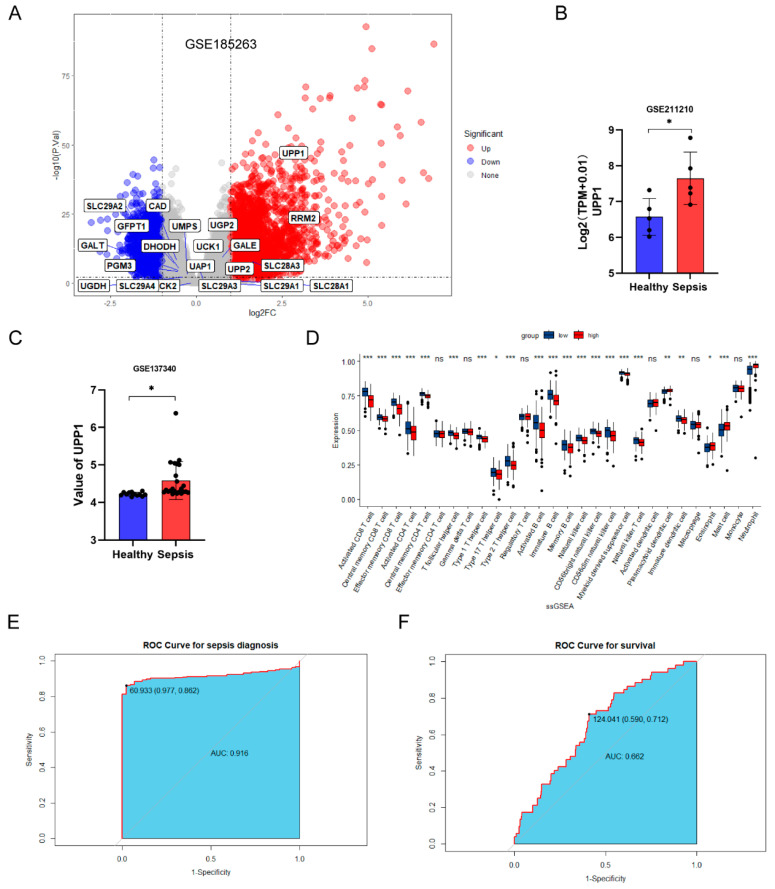
Dysregulated uridine metabolism was confirmed in sepsis patients through meta-GEO datasets. (**A**) The volcano plot for differentially expressed genes in blood samples between sepsis and healthy control in GSE185263. Uridine metabolism-related genes were marked in the plot. (**B**,**C**) Relative UPP1 expression in GSE211210 and GSE137340, respectively. (**D**) Differential analysis of 28 immune-infiltrating cells according to ssGSEA analysis between UPP1 highly expressed and lowly expressed group in patients with sepsis. (**E**,**F**) The receiver operating characteristic (ROC) curve for diagnosing sepsis and predicting survival for patients with sepsis, respectively. (* *p* < 0.05, ** *p* < 0.01, *** *p* < 0.001, ns, no significance).

To confirm the role of uridine metabolism in patients with sepsis, we collected GEO cohorts including human blood samples from healthy individuals and sepsis patients. Differentially expressed analysis showed that uridine metabolism was indeed dysregulated in sepsis and upregulated UPP1 was also verified in other two GEO datasets (Figure 7A–C). Furthermore, we carried out immune infiltration analysis based on ssGSEA algorithm. It was obvious that, in contrast to the UPP1 highly expressed group, the lower neutrophils infiltration was seen in the UPP1 less expressed group (Figure 7D). In addition, as shown in Figure 7D, most immune cells possessed lower infiltration level in the UPP1 highly expressed group, which reflected that UPP1 may serve an inducer for the formation of sepsis-induced immunoparalysis state [32,33]. Next, to explore the ability of UPP1 to diagnose sepsis and predict survival of patients with sepsis, we drew receiver operating characteristic (ROC) curve and calculated area under curve (AUC). The results demonstrated that UPP1 possessed good diagnostic performance (AUC = 0.916) and prognostic prediction performance (AUC = 0.662) for patients with sepsis (Figure 7E,F). To sum up, these bioinformatic analysis further strengthened the notion that uridine metabolism is indeed dysregulated in sepsis and uridine supplementation may be a potent effective avenue for treating sepsis and sepsis-induced ALI.

## 3. Discussion

**Figure 8:**
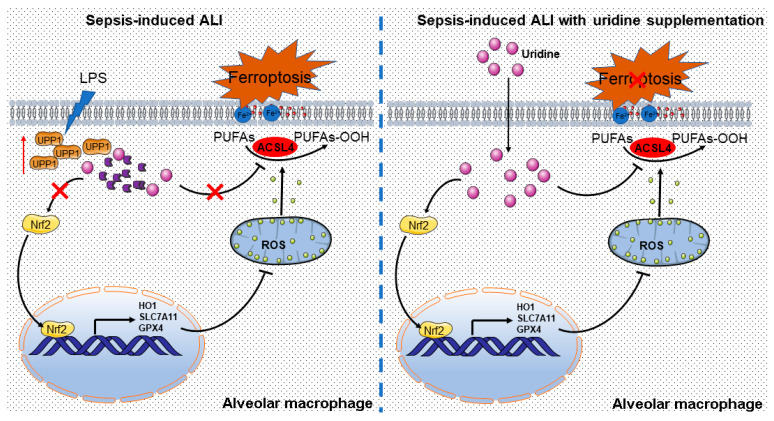
Graphical abstract for uridine supplementation alleviates sepsis-induced ALI. The possible mechanisms by which uridine imposes antioxidative effects to protect against ferroptosis in sepsis-induced ALI.

In the circumstance of sepsis-induced ALI, metabolism in macrophage is extensively reprogramming to response to stress signal, thus, to produce inflammation and aggravate lung damage [34,35]. However, uridine metabolism is poorly investigated in such situations. In the present study, we demonstrated that uridine metabolism is disturbed in sepsis-induced ALI, as manifested by the upregulation of UPP1, which meant to degrade uridine. Subsequently, we discovered that uridine supplementation is sufficient to mitigate sepsis-induced ALI via suppressing ferroptosis of macrophage. Mechanistically, uridine treatment promoted Nrf2 expression and resultant increase of Nrf2-dependent antioxidative targeted genes, including SLC7A11, GPX4 and HO1. Moreover, ACSL4 was also suppressed by exogenous uridine supplementation, which further reinforced the protective effect of uridine in combating ferroptosis. Consistent with previous studies [17,36], we also uncovered that proinflammatory factors, such as TNF-α, IL-1β and IL6, were evidently reduced by uridine treatment. Based on our results, it is supposed that uridine could be a mighty endogenous anti-inflammatory metabolite and a promising preventive remedy for intervening sepsis-induced ALI (Figure 8).

Uridine metabolism plays a pivotal role in cellular biochemical processes, as it is intimately connected to cellular other metabolic processes, such as glucoses, lipids and amino acids homeostasis [10]. Herein, we compared the expression of uridine metabolism-related genes in LPS-induced lung tissue and control to acquire the landscape of uridine metabolism in sepsis-induced ALI. Unexpectedly but reasonably, most uridine metabolism-related genes were unchanged but UPP1 was unraveled to raise in ALI group in both GEO datasets, which was confirmed by experimental ALI model. Further, we validated the results in human blood samples from patients with sepsis. As expected, UPP1 was significantly upregulated in samples from sepsis compared to healthy. These data revealed that uridine metabolism is dysregulated in sepsis-induced ALI and it provided rationale for uridine supplementation to treat sepsis-induced ALI. Actually, a similar situation existed in the context of osteoarthritis, in which UPP1 was upregulated and uridine was decreased, and uridine treatment attenuated the severity of osteoarthritis [37]. Moreover, restraining UPP1 expression to stable uridine homeostasis was reported to prevent progression of liver fibrosis [38,39]. Therefore, we speculated that restoring uridine homeostasis by exogenous supplementation might also be a promising intervention for sepsis-induced ALI.

Subsequently, to test the hypothesis, we treated mice with uridine when sepsis-induced ALI was built. Reassuringly, uridine supplementation observably alleviated lung injury in vivo and refrained macrophage inflammation in vitro. This is consistent with previous studies that the systematic inflammation was strongly inhibited by uridine treatment and this further solidified the fact that uridine possesses anti-inflammatory effect [23].

Ferroptosis is a regulated cell death mediated by redox imbalance, which is intimately regulated by Nrf2 and its downstream targeted genes [29]. Several lines of study have revealed that ferroptosis serves a pathogenic role in sepsis-induced ALI [40,41,42]. Uridine was extensively reported to exhibit antioxidant effect and regarded as an antioxidative metabolite [18,43]. For example, it has been covered that uridine converted to uridine diphosphate (UDP) and subsequently activated mitoKATP to suppress ROS production in myocardial tissue [21]. Thus, we explored the relationship between uridine metabolism and ferroptosis under the context of ALI in vivo and vitro. It was found that uridine treatment upregulated Nrf2 and its targeted genes and increased GSH level and reduced lipid peroxidative product MDA in lung tissue and macrophage. Moreover, the iron level in lung tissue was markedly lowered. Furthermore, Nrf2 silence abrogated the protective effect of uridine. Our results suggested that uridine might suppress ferroptosis via activating antioxidant system through boosting Nrf2 expression. Of note, the lipid synthesis gene ACSL4 was found to be suppressed after uridine supplementation. It is well characterized that ACSL4 promotes polyunsaturated fatty acids synthesis and facilitates ferroptosis [44,45]. Our data prompted that LPS stimulation upregulated ACSL4 expression, while was prevented by uridine supplementation. It may be explained that uridine promotes cell energy excess [46], thus, to boost lipidolysis and inhibit lipid synthesis, which is still deserving of investigation.

Finally, there are some limitations in this study. In our study, although we disclosed that uridine prevented sepsis-induced ALI via controlling ferroptosis, it should not be ignored that uridine exerted anti-inflammatory effects by means of other pathways, such as nuclear transcription factor-κB (NF-κB) pathway [47]. Moreover, the mechanism for uridine to activate Nrf2 is not explored in this study, which needs further study. Furthermore, we mainly investigated the relationship between uridine and ferroptosis in the perspective of lipid peroxidation, but it is also meaningful to explore the relationship between uridine metabolism and iron metabolism.

## 4. Methods and Materials

### 4.1. Chemical and Reagents

Uridine (HY-B1449) was purchased from MedChem Express (Shanghai, China), purity > 99.99%. Lipopolysaccharides (LPS) and Escherichia coli O55:B5 (#L2880) was purchased from Sigma-Aldrich (St Louis, MO, USA). Primary antibodies against Beta Actin (66009-1-Ig), SLC7A11 (26864-1-AP), GPX4 (67763-1-Ig), HO-1 (10701-1-AP), Nrf2 (16396-1-AP) were purchased from Proteintech (Wuhan, China). Anti-ASCL4 (A20414) was purchased from Abclonal Technology (Wuhan, China). Secondary antibodies (GB23301, GB23303) were purchased from Servicebio (Wuhan, China). Malondialdehyde (MDA) assay kit (TBA method) (A003-1-2) and tissue iron assay kit (A039-2-1) were purchased from Jiancheng Bioengineering Institute (Nanjing, China). MPO activity assay kit was purchased from Abcam (Cambridge, UK). ELISA kits for Tumor necrosis factor-α (TNF-α), Interleukin-1beta (IL-1β) and Interleukin 6 (IL6) were acquired through Cloud-Clone (Wuhan, China). The DCFH-DA probe, CCK-8 assay kit, JC-1 probe and Glutathione Peroxidase (GSH-PX) assay kit were obtained from Beyotime Biotechnology (Shanghai, China). The C11-BODIPY 581/591 was purchased from Invitrogen (Waltham, MA, USA).

### 4.2. Establishment of Sepsis-Induced Acute Lung Injury Model

All the animal procedures conducted in the present study conformed to the guidelines of the Care and Use of Laboratory Animals and approved by the Animal Use Committees of Renmin Hospital of Wuhan University. Male C57BL/6J mice (6–8 weeks old and 18–21 g weight) were provided by Hubei Province Experimental Animal (Wuhan, Hubei, China) and fed in appropriate temperature and humidity, with free access to standard food and water, in a room with a 12 h light:12 h dark cycle. All animals were raised in a specific-pathogen free (SPF) environment. Mice were randomly divided into four groups: PBS group, uridine group, LPS group and LPS+uridine group. Unless stated otherwise, the number of animals in each group was 6. Intraperitoneal LPS injection (10 mg/kg) was employed to establish sepsis-induced ALI model according to previous study [48]. Uridine dissolved in PBS was intraperitoneally injected as soon as possible after LPS injection. LPS simulation and uridine administration was simultaneously proceeded. After stimulating with LPS 12 h, animals were sacrificed. Bronchoalveolar lavage fluid (BALF) and lung tissues were collected for further analysis.

### 4.3. BALF Collection, Processing and Determination of Protein Concentration

For BALF collection, the cervical trachea was exposed, and 1 mL phosphate-buffered sterile saline was instilled into alveoli via the trachea by means of a syringe. After rinsing the lungs three times with the recovered lavage fluid, final BALF was collected. Subsequently, the BALF was centrifuged at 800 g for 10 min, and the protein level in the supernatant was determined by bicinchoninic acid (BCA) protein assay kit (Beyotime, Shanghai, China).

### 4.4. Lung Wet-to-Dry Weight Ratio Measurement

To assess lung tissue edema, the right lungs were cleaned and weighed to obtain the wet weight. Then the lungs were kept at 70 °C for 48 h in an incubator. Lungs were then weighed to obtain the dry weight. The wet-to-dry (W/D) weight ratio of the lung was calculated.

### 4.5. H&E Staining and Lung Injury Score Evaluation

Left lung tissues were fixed with 10% buffered formalin, embedded in paraffin, and then cut into slices with thickness about 4 μm. Subsequently, the lung sections were stained with hematoxylin and eosin (H&E) (Servicebio, Wuhan, China) and observed under a microscope. The severity of lung injury was scored according to published criteria [49].

### 4.6. Assay of MPO Activity

Myeloperoxidase (MPO) activity assay kit (#ab105136, Abcam) was used to detect the MPO activity of lung tissue homogenate. The absorbance at 412 nm was determined through a microplate reader.

### 4.7. Quantification of Cytokines, Detection of Tissue Iron and Lipid Peroxidation Level

TNF-α, IL-1β, and IL-6 were quantified by following the ELISA kit instructions. Tissue iron concentrations determination was implemented by following the specification. The level of lipid peroxidation was measured by MDA assay kit as instructed.

### 4.8. Cell Culture and Transfection

The human monocytic leukemia cell line (THP-1), the human-type II cell alveolar epithelial cells (A549) and human umbilical vein endothelial cells (HUVEC) were purchased from the American Type Culture Collection (ATCC, Manassas, VA, USA). THP-1 cells were cultured in RPMI 1640 culture medium (Servicebio, Wuhan, China). A549 cells were cultured in F12K medium and HUVEC was cultured in DMEM medium with high glucose. All the culture media were supplemented with 10% fetal bovine serum (FBS; Gibco, Waltham, MA, USA) and 1% penicillin/streptomycin (P/S) (Biosharp, Hefei, China). Cells were maintained at 37 °C, 5% CO2 humidified incubator. For Nrf2 knockdown assay, the THP-1 cells were transfected with Nrf2 siRNA (5′-AUUGAUGUUUCUGAUCUAUCACUTT-3′) using Lipofectamine 2000 (Invitrogen, Waltham, MA, USA) according to the instruction.

### 4.9. Macrophage Differentiation and Treatment

The THP-1 monocytes were stimulated with 100 ng/mL 1 phorbol 12-myristate 13-acetate (PMA; MCE) for 48 h to acquire macrophage-like state. Then, cells were digested using trypsin and centrifuged and resuspended with culture medium and then seeded into 6-wells cell culture plates (Corning, Corning, NY, USA). After PMA induced, differentiated macrophage-like THP-1 cells were cultured in RPMI 1640 medium without PMA but containing 1% PBS and 10% FBS for 12 h. After that, cells were treated with uridine or LPS or both. The concentrations for uridine were 50 μM, 100 μM, 200 μM in the initial cell experiment for selecting appropriate concentration and eventually 200 μM was selected as the optimum. As for LPS, unless stated, 10 μg/mL was used to stimulate. In experiments, we treated uridine simultaneously after LPS stimulation.

### 4.10. GSH Measurement

Glutathione Peroxidase (GSH-PX) assay kit was used to detect the total GSH in cells and tissue according to the instruction.

### 4.11. Cell Viability Assay

Cell Counting Kit-8 (CCK-8) assay was used to detect the cell viability. In brief, 10 μL CCK-8 solution was added into the 96-well plates and incubated for an hour at incubator. The absorbance was examined at 450 nm according to CCK8 kit instruction.

### 4.12. RNA Extraction and Real-Time Polymerase Chain Reaction

The total RNA of lung tissues and THP-1 cells were extracted using TRIpure Total RNA Extraction Reagent (Biosharp, China), and cDNA was synthesized using cDNA Synthesis Kit (Servicebio, Wuhan, China). Quantitative real-time PCR was performed using SYBR Green PCR SuperMix (Servicebio, China). The relative expression of target genes unified to Actin were calculated. All primers used in this study were listed in Table 1.

### 4.13. Western Blotting

Protein extraction from cells or tissues were performed by the lysis of RIPA Lysis Buffer (Servicebio, Wuhan, China) contained with 1% Phenylmethanesulfonyl fluoride (PMSF, Servicebio, Wuhan, China). Protein separation was proceeded on 10–15% SDS-polyacrylamide gradient gels and PVDF membranes were used for proteins transmembrane. Protein Free Rapid Blocking Buffer (5×) was used to block non-specific binding for 15 min, and membranes were incubated with primary antibodies in 4 °C for overnight. Then, membranes were washed three times with TBST, each time for 10 min. Subsequently, membranes were incubated with anti-rabbit-HRP (1:2000; Servicebio) or anti-mouse-HRP (1:2000; Servicebio, Wuhan, China) in 37 °C for 1 h. The internal reference protein was Actin. The protein bands were exposed by the enhanced chemiluminescence western blotting detection system (Bio-Rad, Hercules, CA, USA).

### 4.14. ROS, Lipid Peroxidation and Mitochondrial Potential Measurement

The DCFH-DA probe was used to determine the reactive oxygen species (ROS) level of THP-1 cells. The C11 BODIPY was used to detect the lipid peroxidation level of THP-1 cells. The JC-1 probe was used to detect mitochondrial potential of THP-1 cells. All the operational processes were performed according to instructions and fluorescence intensity was detected by fluorescence microscopy.

### 4.15. Dataset Collection and Preprocessing

Uridine metabolism-related genes were collected from previously published literatures. GEO cohorts (GSE130936, GSE2411, GSE15379, GSE1871, GSE185263, GSE211210, GSE137340) and related platform files were downloaded from GEO database (https://www.ncbi.nlm.nih.gov/geo/ (accessed on 15 September 2022)). The probes were transformed to gene symbols via corresponding platform annotation files. GSE130936 and GSE2411 were integrated into a new cohort using the ‘ComBat’ algorithm of ‘sva’ package to reduce the batch effects. GSE15379 and GSE1871 were also doing the same. The information about the GEO cohorts in the present study were listed in Table 2.

### 4.16. Bioinformatic Analysis and Visualization

The limma package was used to identify the differentially expressed genes. The ggplot2 package was used to visualize the results from limma through drawing volcano plots. STRING website and Cytoscape software were used to construct protein–protein interaction (PPI) network. Gene ontology (GO) analysis was implemented in Metascape online website. ssGSEA algorithm of GSVA package was used to implement immune infiltration analysis. The Receiver Operating Characteristic (ROC) curves were drawn by pROC package and area under curve (AUC) were calculated.

### 4.17. Statistical Analysis

All statistical analyses were performed with GraphPad Prism 9 software and R software. Data were expressed as mean ± standard deviation (SD). Two group comparison were performed using the Student’s *t*-test with Welch’s correction. While for multiple group comparisons, the one-way ANOVA with the post hoc Tukey test was performed. Unless stated otherwise, the data represent at least three independent experiments. *p* < 0.05 was regarded as statistically significant.

## 5. Conclusions

In summary, we described the dysregulated uridine metabolism in sepsis-induced ALI for the first time and discovered that uridine supplementation could inhibit ferroptosis of macrophage via Nrf2 pathway and ACSL4 inhibition. It provides a novel insight and rationale for targeting UPP1 by specific inhibitor or uridine supplementation for clinical use to combat sepsis-induced ALI.

## Figures and Tables

**Table 1:** The primers used in real-time PCR.

Species	Gene	Forward Primer	Reverse Primer
Mice	TNF-α	ACTGAACTTCGGGGTGATCGGT	TGGTTTGCTACGACGTGGGCTA
Mice	IL-6	CCCCAATTTCCAATGCTCTCC	CGCACTAGGTTTGCCGAGTA
Mice	IL-1β	AATGAAGGAACGGAGGAGCC	CTCCAGCCAAGCTTCCTTGT
Mice	GPX4	GCTGGGAAATGCCATCAAAT	TCCTTCTCTATCACCTGGGGCT
Mice	SLC7A11	GAAATATCAGGTCATTGGTGGAGA	ATGCTCCTGCTTGAGTATGTCG
Mice	HO1	GCCAGCAACAAAGTGCAAGA	TAAGGACCCATCGGAGAAGC
Mice	UPP1	ATCCCAACATCTGTGCAGGC	ACCTGGCATGGTACAGCATC
Mice	β-actin	GTCCACCGCAAATGCTTCTA	TGCTGTCACCTTCACCGTTC
Human	TNF-α	TCCAGGCGGTGCTTGTTC	GCTTGTCACTCGGGGTTC
Human	IL-6	ATGAGGAGACTTGCCTGGTGAA	CTCTGGCTTGTTCCTCACTACTCTC
Human	IL-1β	CGGGACTCACAGCAAAAAA	TTCAACACGCAGGACAGGT
Human	GPX4	TGAAGATCCAACCCAAGGGC	GACGGTGTCCAAACTTGGTG
Human	SLC7A11	GGCAGTTGCTGGGCTGATTTA	GATGACGAAGCCAATCCCTGT
Human	HO1	ATGCCCCAGGATTTGTCAGAG	GGAAGTAGACAGGGGCGAAGAC
Human	UPP1	CACCACTAGCAGACACAATTTCC	AATGCCCATACCATGACTGACAG
Human	β-actin	CACCCAGCACAATGAAGATCAAGAT	CCAGTTTTTAAATCCTGAGTCAAGC

**Table 2:** The information of GEO datasets.

Dataset Number	Platform	LPS Group/Sepsis	Control Group/Healthy	Annotation	Sample Type
GSE130936	GPL339	6	4	Affymetrix Mouse Expression 430A Array	Murine lung
GSE15379	GPL1261	3	3	Affymetrix Mouse Genome 430 2.0 Array	Murine lung
GSE2411	GPL339	6	6	Affymetrix Mouse Expression 430A Array	Murine lung
GSE1871	GPL1261	3	3	Affymetrix Mouse Genome 430 2.0 Array	Murine lung
GSE211210	GPL24676	5	5	Illumina NovaSeq 6000 (Homo sapiens)	Human Blood
GSE137340	GPL10558	45	12	Illumina HumanHT-12 V4.0 expression beadchip	Human Blood
GSE185263	GPL16791	348	44	Illumina HiSeq 2500 (Homo sapiens)	Human Blood

## Data Availability

All data that support the findings in this study are available from the corresponding author upon reasonable request.
